# Altered Metal Homeostasis Associates with Inflammation, Oxidative Stress, Impaired Glucose Metabolism, and Dyslipidemia in the Crosstalk between Childhood Obesity and Insulin Resistance

**DOI:** 10.3390/antiox11122439

**Published:** 2022-12-10

**Authors:** Álvaro González-Domínguez, María Millán-Martínez, Jesús Domínguez-Riscart, Rosa María Mateos, Alfonso María Lechuga-Sancho, Raúl González-Domínguez

**Affiliations:** 1 Instituto de Investigación e Innovación Biomédica de Cádiz (INiBICA), Hospital Universitario Puerta del Mar, Universidad de Cádiz, 11009 Cádiz, Spain; 2 Associate Unit CSIC-University of Huelva “Atmospheric Pollution”, Center for Research in Sustainable Chemistry—CIQSO, University of Huelva, 21071 Huelva, Spain; 3 Department of Chemistry, Faculty of Experimental Sciences, University of Huelva, 21071 Huelva, Spain; 4 Unidad de Endocrinología Pediátrica y Diabetes, Servicio de Pediatría, Hospital Universitario Puerta del Mar, 11009 Cádiz, Spain; 5 Área de Bioquímica y Biología Molecular, Departamento de Biomedicina, Biotecnología y Salud Pública, Universidad de Cádiz, 11519 Cádiz, Spain; 6 Departamento Materno Infantil y Radiología, Facultad de Medicina, Universidad de Cádiz, 11009 Cádiz, Spain

**Keywords:** childhood obesity, metals, inflammation, oxidative stress, insulin resistance, erythrocyte

## Abstract

Metals are redox-active substances that participate in central biological processes and may be involved in a multitude of pathogenic events. However, considering the inconsistencies reported in the literature, further research is crucial to disentangle the role of metal homeostasis in childhood obesity and comorbidities using well-characterized cohorts and state-of-the-art analytical methods. To this end, we studied an observational population comprising children with obesity and insulin resistance, children with obesity without insulin resistance, and healthy control children. A multi-elemental approach based on the size-fractionation of metal species was applied to quantify the total content of various essential and toxic elements in plasma and erythrocyte samples, and to simultaneously investigate the metal fractions conforming the metalloproteome and the labile metal pool. The most important disturbances in childhood obesity were found to be related to elevated circulating copper levels, decreased content of plasmatic proteins containing chromium, cobalt, iron, manganese, molybdenum, selenium, and zinc, as well as the sequestration of copper, iron, and selenium within erythrocytes. Interestingly, these metal disturbances were normally exacerbated among children with concomitant insulin resistance, and in turn were associated to other characteristic pathogenic events, such as inflammation, oxidative stress, abnormal glucose metabolism, and dyslipidemia. Therefore, this study represents one-step further towards a better understanding of the involvement of metals in the crosstalk between childhood obesity and insulin resistance.

## 1. Introduction

Childhood obesity is a multi-factorial disorder associating a myriad of complications, including abnormal carbohydrate metabolism, dyslipidemia, chronic low-grade inflammation, and oxidative stress [1]. In this respect, growing evidence points to metal and metalloid elements as potential regulators of these heterogeneous disturbances. Metals play pivotal roles in a wide range of essential biological processes, such as electron and oxygen transport, immune response, synthesis of fatty acids, proteins, RNA and DNA, cell division, antioxidant defense, hormonal regulation, and many others [2,3,4]. Accordingly, impaired metal homeostasis may considerably impact the proper functioning of central metabolic pathways and thus contribute to the development of a variety of diseases, including obesity and related comorbidities. To date, only a few authors have investigated the relationship between childhood obesity and alterations in levels of essential and toxic elements in different biological matrices, namely whole blood [5,6], serum/plasma [6,7,8,9,10], urine [6,11,12], hair [13], and teeth [5]. In general, excess body mass has been associated with deficiencies of magnesium, zinc, iron, and selenium, as well as with elevated copper levels, while contradictory results have been reported for other minor elements. However, it should be noted here that all these previously published studies relied on the measurement of total metal contents in the biological samples under investigation, without taking into consideration the chemical form in which trace elements are present in the organism. The main biological function of metals results from their incorporation as enzymatic cofactors into the structure of metalloproteins, although they can also be detected as free labile ions or in the form of metallometabolites. As this distinction between high molecular mass (HMM) and low molecular mass (LMM) species determines their biological activities, toxicological properties, and transport across biological compartments [14], characterizing the multi-elemental biodistribution is essential to better understand the role of metals in health status. On the other hand, another major limitation of the available literature on this topic is related to the study population. Despite that obesity can normally be accompanied by various comorbidities, such as insulin resistance (IR), dyslipidemia, or glucose intolerance, all the studies reviewed above considered it as a homogeneous disorder. However, part of the obese population (ca. 10–30%) is not affected by these metabolic disturbances and paradoxically presents low cardiometabolic risk, which constitutes the “metabolically healthy obesity” [15]. Altogether, further investigation of the multi-elemental status in well-characterized cohorts seems to be crucial to obtain new insights into the involvement of metals in childhood obesity and its metabolic complications.

Herein, we investigated the multi-elemental profiles in peripheral blood samples from a case-control study design comprising children with obesity and IR, children with obesity without IR, and healthy control children. To this end, a high-throughput metallomics method was employed for the first time for the size-fractionation of circulating metal species. The methodology was applied to plasma and erythrocyte samples to obtain a comprehensive and multi-compartmental overview of the characteristic alterations in metal homeostasis behind childhood obesity and IR, and to decipher their association with concomitant pathogenic events such as hyperglycemia, hyperinsulinemia, dyslipidemia, inflammatory processes, and exacerbated oxidative stress. In this vein, the study of the erythroid fraction could provide added value with respect to the available scientific evidence in serum/plasma, especially considering that erythrocytes are powerful systemic indicators of the metabolic and redox status of the organism [16].

## 2. Materials and Methods

### 2.1. Study Population and Sample Collection

The study population consisted of prepubertal children (Tanner Stage I) aged between 6 and 10 years, comprising children with obesity and IR (ObIR+, *n* = 31), children with obesity without IR (ObIR−, *n* = 15), and healthy control children (CNT, *n* = 26). Children with obesity were recruited among patients who attended the Pediatric Endocrinology Unit of “Hospital Universitario Puerta del Mar” (Cádiz, Spain) for routinary health assessment. These study participants did not receive any specific treatment (e.g., medication, lifestyle guidance) along the consecution of the research project. Children with a body mass index (BMI) over two standard deviations above the mean of the reference population, adjusted for sex and age, were diagnosed as obese [17]. When indicated by clinical findings, children with obesity underwent an oral glucose tolerance test (OGTT), and the measurement of blood insulin levels along the OGTT curve enabled us to diagnose concomitant IR when meeting at least one of the following criteria: (i) homeostasis model assessment of insulin resistance (HOMA-IR) score above 3.5, (ii) fasting insulin levels above 15 mU/L, (iii) insulin levels above 75 mU/L at 120 min of the OGTT, (iv) insulin levels above 150 mU/L at any time point of the OGTT [18]. On the other hand, healthy non-obese children who needed a blood test for other medical reasons (e.g., pre-anesthesia) were enrolled as control subjects. From all participants, venous blood samples were collected in the morning after overnight fasting. Blood tubes were centrifuged at 1500× *g* for 10 min at 4 °C to separate the plasma. The resulting pellets were then washed three times with cold saline solution (9 g/L NaCl, 4 °C) to obtain the erythrocyte fraction by centrifuging at 1500× *g* for 5 min at 4 °C. Plasma and erythrocyte samples were aliquoted and stored at −80 °C until analysis. The study was performed in accordance with the principles contained in the Declaration of Helsinki. The Ethical Committee of “Hospital Universitario Puerta del Mar” (Cádiz, Spain) approved the study protocol (Ref. PI22/01899), and all participants and/or legal guardians provided written informed consent.

### 2.2. Anthropometric and Biochemical Variables

Anthropometric variables, including weight, height, BMI, and waist circumference (WC), were evaluated by pediatric endocrinologists. Routine clinical assays were employed to determine fasting plasma levels of glucose, insulin, glycated hemoglobin (HbA1c), C-reactive protein (CRP), and lipid profile (i.e., total cholesterol, TC; high-density lipoprotein cholesterol, HDL-C; low-density lipoprotein cholesterol, LDL-C; triglycerides, TG). Additionally, glucose and insulin concentrations were also measured along the OGTT curve in the plasma of children with obesity (i.e., 0, 30, 60, 90, 120 min). The homeostasis model assessment of insulin resistance (HOMA-IR), the whole-body insulin sensitivity index (WBISI), the area under the curve for glucose (AUC_Glc_), the area under the curve for insulin (AUC_Ins_), and the Castelli risk index-I (CRI-I) were calculated by applying the following formulas [19]:

HOMA-IR = (Glc_0_ × Ins_0_) × 0.055/22.5 (1)

WBISI = 10,000/(Glc_0_ × Ins_0_ × MeanGlc × MeanIns)^1/2^ (2)

AUC_Glc_ = 0.25 × Glc_0_ + 0.5 × Glc_30_ + 0.75 × Glc_60_ + 0.5 × Glc_120_ (3)

AUC_Ins_ = 0.25 × Ins_0_ + 0.5 × Ins_30_ + 0.75×Ins_60_ + 0.5 × Ins_120_ (4)

CRI-I = TC/HDL-C(5)

where Glc_0_ and Ins_0_ refer to the fasting plasma concentrations of glucose and insulin; Glc_30_, Glc_60_, and Glc_120_ to the plasma glucose concentrations measured at 30, 60, and 120 min along the OGTT; Ins_30_, Ins_60_, and Ins_120_ to the plasma insulin concentrations measured at 30, 60, and 120 min along the OGTT; MeanGlc and MeanIns to the mean glucose and insulin concentrations, respectively, measured in plasma along the OGTT. Glucose and insulin concentrations are expressed as mg/dL and µU/mL, respectively.

White blood cell count measurements were performed using an automated hematology analyzer and subsequently employed to compute different inflammatory indices, namely the systemic immune inflammation index (SII), the systemic inflammation response index (SIRI), the aggregate index of systemic inflammation (AISI), the platelet-to-lymphocyte ratio (PLR), and the neutrophil-to-lymphocyte ratio (NLR), using the following formulas:

SII = N × P/L(6)

SIRI = N × M/L(7)

AISI = N × P × M/L(8)

NLR = N/L(9)

PLR = P/L(10)

where, N, P, L, and M represent neutrophil counts, platelet counts, lymphocyte counts, and monocyte counts, respectively.

Finally, various oxidative stress markers were assessed in erythrocyte samples using the spectrophotometric methods described elsewhere for determining the contents of thiobarbituric acid reactive substances (TBARS) and protein carbonyls (PC), as well as the catalase (CAT) activity [19].

### 2.3. Multi-Elemental Analysis of Plasma and Erythrocyte Samples

For the analysis of the total metal contents, aliquots of plasma (150 µL) and erythrocyte (50 µL) samples were diluted to a final volume of 3 mL using an alkaline solution containing 2% 1-butanol (*w*/*v*), 0.05% EDTA (*w*/*v*), 0.05% Triton X-100 (*w*/*v*), and 1% NH_4_OH (*w*/*v*) [20]. Complementarily, samples were also subjected to protein precipitation under non-denaturing conditions for size-fractionation of metal species following the methodology optimized by González-Domínguez et al. [21,22]. Briefly, 300 µL of cold acetone (−20 °C) was added dropwise to 150 µL of plasma, or 50 µL in the case of erythrocytes. The samples were vortexed for 10 min at 4 °C using an orbital rotator mixer, and subsequently centrifuged at 10,000× *g* for 10 min at 4 °C. Then, the supernatant was transferred to a new tube and taken to dryness using a SpeedVac system (Cole-Parmer, Vernon Hills, IL, USA). Finally, the dried supernatants (i.e., LMM metal species) and the protein pellets (i.e., HMM metal species) were reconstituted in 3 mL of the alkaline solution described above by sonicating for 10 min. Quality control (QC) samples were prepared by pooling equal volumes of each sample under study and treated as the rest of the samples. Rhodium (internal standard) was added to all the sample extracts (i.e., Total, HMM, LMM) to reach a final concentration of 1 µg/L. Before analysis, samples were filtered through 0.45 µm pore size hydrophilic PTFE filters.

Multi-elemental analyses were performed in an Agilent 7900 inductively-coupled plasma mass spectrometer (ICP-MS) equipped with collision/reaction cell system and with nickel sampling and skimmer cones (Agilent Technologies, Tokyo, Japan). High-purity grade helium (>99.999%) was employed as the collision gas. Instrumental conditions were optimized using a tuning solution containing 1 μg/L lithium, cobalt, yttrium, and thallium. The ICP-MS operating conditions were set as follows [22]: sampling depth, 7 mm; forward power, 1550 W; plasma gas, 15 L/min; auxiliary gas, 1 L/min; carrier gas, 1 L/min; make-up gas, 0.10 L/min; helium, 5 mL/min. The isotopes monitored were ^51^V, ^52^Cr, ^53^Cr, ^55^Mn, ^56^Fe, ^57^Fe, ^59^Co, ^63^Cu, ^66^Zn, ^77^Se, ^78^Se, ^82^Se, ^95^Mo, ^98^Mo, ^103^Rh, ^111^Cd, and ^208^Pb, using a dwell time of 0.3 s per isotope. For quantification purposes, multi-elemental calibration curves were prepared in alkaline solution within the concentration range 0.5–2500 µg/L, containing 1 µg/L rhodium as the internal standard. Within each batch (i.e., plasma-Total, plasma-HMM, plasma-LMM, erythrocytes-Total, erythrocytes-HMM, erythrocytes-LMM), samples were analyzed in random order and one QC sample was intercalated every 10 samples. Blank samples were analyzed at the beginning and at the end of each sequence run.

### 2.4. Statistical Analysis

Clinical and biochemical characteristics were compared by analysis of variance (ANOVA) followed by the Fisher LSD post-hoc test. Metallomics data pre-processing and analysis were performed with the MetaboAnalyst 5.0 web tool (https://www.metaboanalyst.ca/, accessed on 28 October 2022), as follows. First, variables with more than 20% missing values were removed, and the remaining missing values were imputed using the kNN algorithm. Then, the data were log transformed and Pareto scaled. To look for differences between the study groups, data were subjected to ANOVA with Fisher LSD post hoc test, adjusted for multiple comparisons using the Benjamini-Hochberg false discovery rate (FDR). FDR-corrected p-values below 0.05 were considered statistically significant. Furthermore, Pearson’s correlations were computed between metal levels and other anthropometric and biochemical variables, including parameters related to obesity, glucose and lipid metabolism, inflammation, and oxidative stress.

## 3. Results

### 3.1. Clinical and Biochemical Characterization of the Study Population

The study groups were similar in age and sex distribution, whereas obesity-related clinical parameters (i.e., weight, BMI, WC) were evidently higher in children with obesity, regardless the concomitant presence or absence of IR (Table 1). Children with obesity also showed elevated plasma levels of fasting insulin, with increased HOMA-IR scores among IR subjects. As expected, OGTT-related variables were different between the two obesity sub-samples, being the ObIR+ study group characterized by higher area under the curve for insulin and mean concentrations of glucose and insulin, as well as by decreased WBISI scores. In turn, this hyperglycemic status was reflected in increased content of glycated hemoglobin in the plasma of children with obesity, as reported elsewhere [23]. Regarding lipid metabolism, cases presented lower HDL-C content, and increased triglyceride levels and Castelli risk index-I scores. Childhood obesity, especially when accompanied by IR, was also characterized by raised inflammatory (i.e., CRP, SII, SIRI, AISI, NLR) and oxidative stress (i.e., TBARS, PC) markers, as well as by impaired antioxidant defense (i.e., reduced catalase activity), in line with previous data [19,23].

### 3.2. Alterations in Plasmatic and Erythroid Multi-Elemental Profiles

The total metal contents measured in the study samples (Table 2) were within the normal concentration ranges previously reported for human plasma and erythrocytes [24], and particularly within those found in other childhood obesity cohorts [6,7,8,9,10]. The application of the size-fractionation method showed that trace elements present in blood were majorly distributed within the HMM fraction, whereas LMM species normally accounted for less than 10% of the total. This is in line with previous studies based on the same analytical methodology conducted in blood samples from patients with Alzheimer’s disease [25,26]. When considering total metal contents, only plasma copper was found to be significantly different between the study groups, displaying higher concentrations in children with obesity, regardless the IR status, compared to controls (Table 2). In contrast, the analysis of the plasmatic metalloproteome (i.e., HMM fraction) corroborated the increase in copper levels in children with obesity, but also a substantial decline in the contents of chromium, cobalt, iron, manganese, molybdenum, selenium, and zinc in ObIR+ individuals and, to a lesser extent, in the ObIR− group. Otherwise, an accumulation of copper, iron, and selenium was observed in the erythroid HMM fraction of children with obesity, especially in those presenting concomitant IR. No significant differences were found in LMM metal species in either plasma or erythrocytes.

### 3.3. Association of Metal Levels with Anthropometric and Biochemical Variables

**Figure 1:**
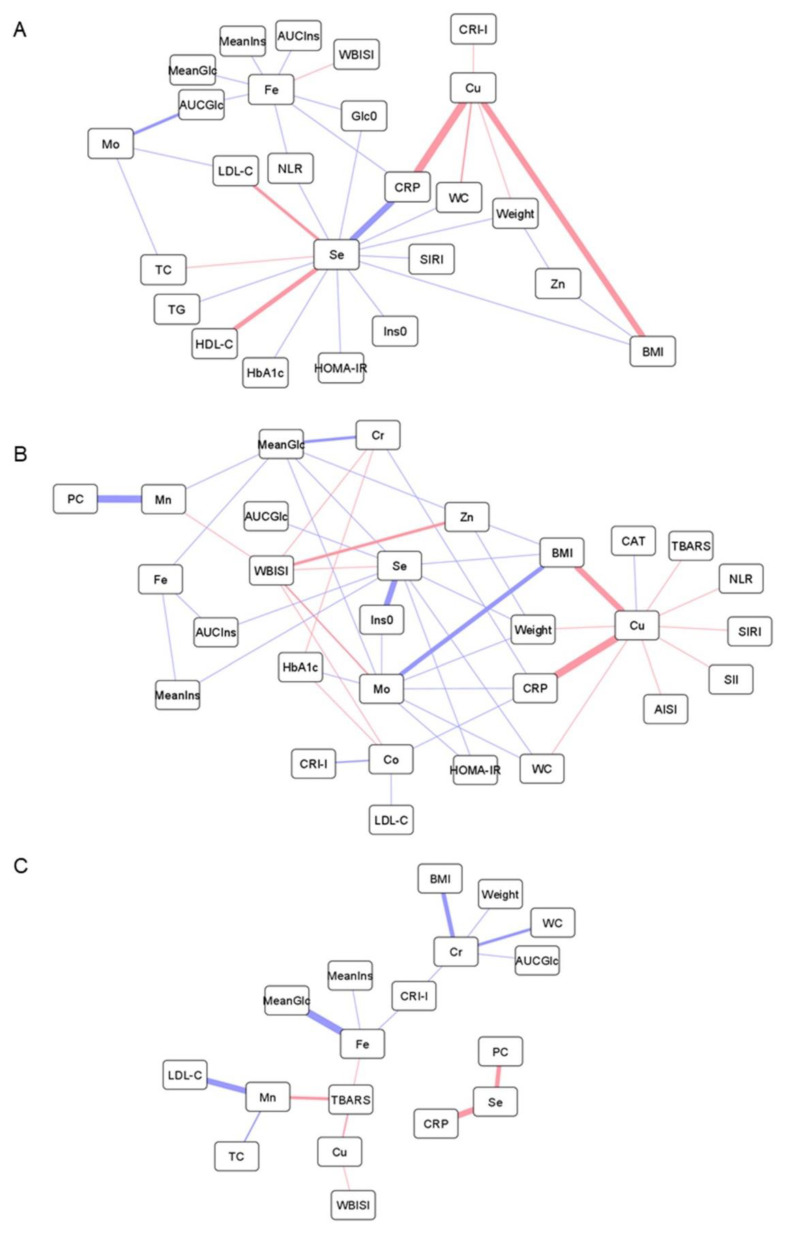
Network representations of Pearson’s correlations between anthropometric and biochemical variables, including parameters related to obesity, inflammation, oxidative stress, glucose and lipid metabolism, and total metal levels in plasma (**A**), HMM metal species in plasma (**B**), and LMM metal species in plasma (**C**). Positive and negative correlations are represented as red and blue lines, respectively (the thicker the line, the stronger the correlation). Abbreviations: AISI, aggregate index of systemic inflammation; AUCGlc, area under the curve for glucose; AUCIns, area under the curve for insulin; BMI, body mass index; CAT, catalase; Co, cobalt; Cr, chromium; CRI-I, Castelli risk index-I; CRP, C-reactive protein; Cu, copper; Fe, iron; Glc0, fasting plasma concentration of glucose; HbA1c, glycated hemoglobin; HDL-C, high-density lipoprotein cholesterol; HOMA-IR, homeostasis model assessment of insulin resistance; Ins0, fasting plasma concentration of insulin; LDL-C, low-density lipoprotein cholesterol; MeanGlc, mean glucose concentration along the oral glucose tolerance test; MeanIns, mean insulin concentration along the oral glucose tolerance test; Mn, manganese; Mo, molybdenum; NLR, neutrophil-to-lymphocyte ratio; PC, protein carbonyl; Se, selenium; SII, systemic immune inflammation index; SIRI, systemic inflammation response index; TBARS, thiobarbituric acid reactive substances; TC, total cholesterol; TG, triglycerides; WBISI, whole-body insulin sensitivity index; WC, waist circumference; Zn, zinc.

**Figure 2:**
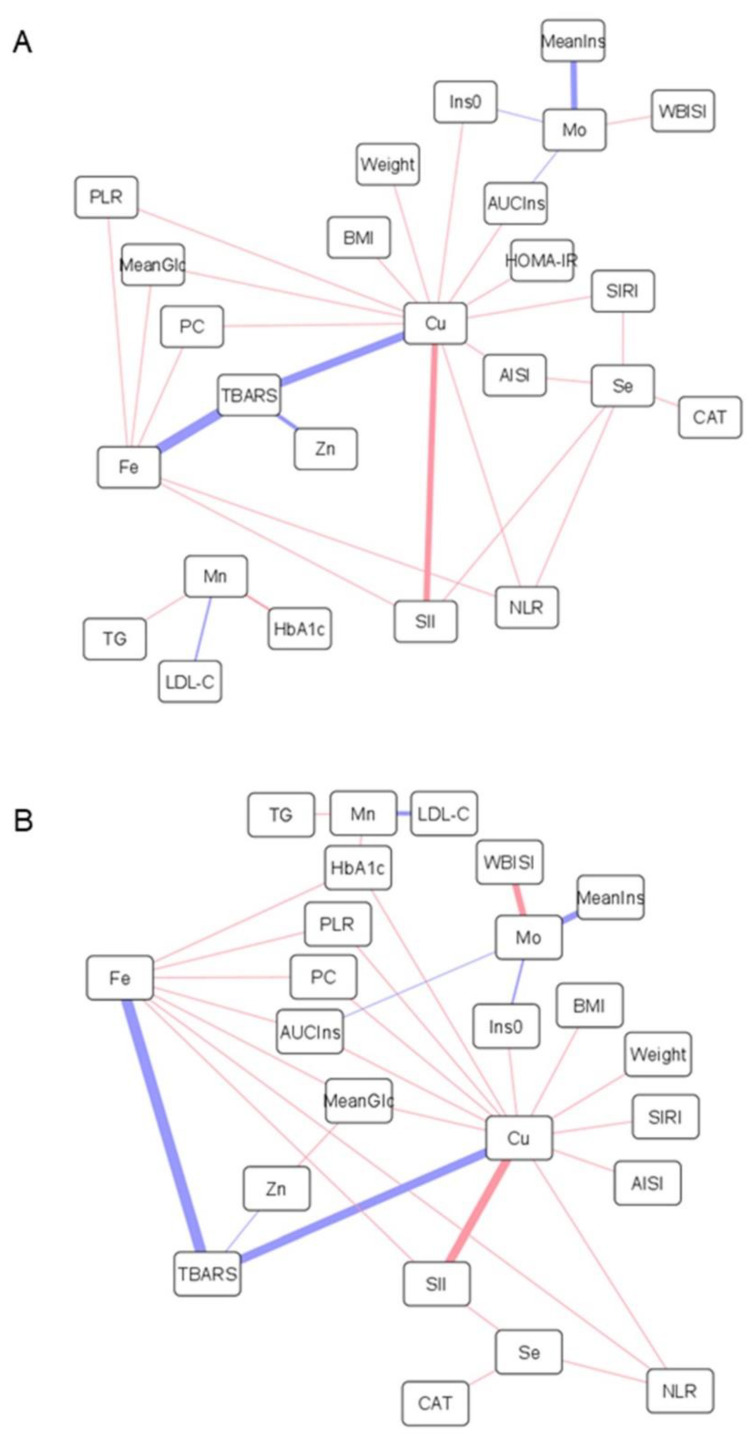
Network representations of Pearson’s correlations between anthropometric and biochemical variables, including parameters related to obesity, inflammation, oxidative stress, glucose and lipid metabolism, and total metal levels in erythrocytes (**A**) and HMM metal species in erythrocytes (**B**). Positive and negative correlations are represented as red and blue lines, respectively (the thicker the line, the stronger the correlation). Abbreviations: AISI, aggregate index of systemic inflammation; AUCIns, area under the curve for insulin; BMI, body mass index; CAT, catalase; Cu, copper; Fe, iron; HbA1c, glycated hemoglobin; HOMA-IR, homeostasis model assessment of insulin resistance; Ins0, fasting plasma concentration of insulin; LDL-C, low-density lipoprotein cholesterol; MeanGlc, mean glucose concentration along the oral glucose tolerance test; MeanIns, mean insulin concentration along the oral glucose tolerance test; Mn, manganese; Mo, molybdenum; NLR, neutrophil-to-lymphocyte ratio; PC, protein carbonyl; PLR, platelet-to-lymphocyte ratio; Se, selenium; SII, systemic immune inflammation index; SIRI, systemic inflammation response index; TBARS, thiobarbituric acid reactive substances; TG, triglycerides; WBISI, whole-body insulin sensitivity index; Zn, zinc.

Correlation analyses were performed to investigate the connection between the alterations found in multi-elemental profiles and other pathogenic hallmarks underlying childhood obesity, including inflammatory processes, oxidative stress, impaired carbohydrate metabolism, and dyslipidemia. As shown in Figure 1 and Figure 2, obesity-related parameters (i.e., weight, BMI, WC) were positively correlated with total and HMM copper contents in both plasma and erythrocytes, whereas negative associations were found with plasma levels of total and HMM-bound zinc and selenium, LMM chromium species, and molybdenum-containing proteins. A positive correlation was found between inflammatory markers (i.e., white blood cell-based inflammatory indices), plasma levels of copper, and erythroid levels of copper, zinc iron, selenium, and manganese (total and HMM fractions). Conversely, the correlation was inverse for total plasma iron, selenium, and manganese, as well as for plasmatic chromium and molybdenum metalloproteins. In the LMM fraction, plasma selenium was positively correlated with CRP. Similarly, oxidative stress markers (i.e., TBARS, PC) were positively associated with cuproproteins and labile pools of copper, iron, selenium, and zinc in plasma, but negatively with Mn-containing proteins. In erythrocytes, the total and HMM fractions of copper, zinc, and iron correlated positively with PC and negatively with TBARS, whereas selenium was positively associated with catalase activity. Regarding carbohydrate metabolism, most of the trace elements measured in plasma showed a protective association against hyperglycemia and hyperinsulinemia, but the correlation was positive for erythroid metals, except for molybdenum. In contrast, HMM chromium and cobalt plasma species were positively associated with glycated hemoglobin. Finally, dyslipidemia-related variables were associated with plasma metal levels as follows: (i) positive correlation with total copper, (ii) negative correlation with HMM species of chromium, cobalt, and molybdenum, and (iii) negative correlation with labile iron and chromium. Plasma selenium showed a positive correlation with cholesterol but negative with triglyceride levels, whereas the opposite trend of association was observed for manganese, in both plasma and erythrocytes.

## 4. Discussion

Although only slight differences were found between the three study groups regarding total metal contents, a deeper investigation of the multi-elemental biodistribution across HMM and LMM fractions illuminated the complex metal dyshomeostasis behind childhood obesity and IR. The application of the size-fractionation methodology evidenced relevant disturbances at the metalloproteome level in children with obesity, whereas other LMM metal species remained unaltered. Further correlation analysis reinforced the central role that trace elements may have in the characteristic metabolic complications and pathological dysfunctions that frequently accompany childhood obesity, as discussed below.

### 4.1. Antagonism between Copper and Zinc

Total and protein-bound copper plasma levels were found to be increased in children with obesity, together with a lower content of zinc-containing HMM species, especially in the ObIR+ group (Table 2). Furthermore, these metals were strongly correlated with obesity parameters (Figure 1A,B, positive correlation for total/HMM copper, negative correlation for total/HMM zinc), suggesting a pivotal role of their homeostasis in childhood obesity and related comorbidities. In this vein, the mechanisms underlying the association between obesity, copper, and zinc could be allocated to the antagonistic interactions between these two essential elements. On the one hand, the characteristic chronic low-grade inflammation observed in obesity, and particularly the secretion of pro-inflammatory cytokines by adipose tissue, is known to induce the expression of zinc transporters [27], which may alter the distribution of zinc in the body and, consequently, reduce the circulating concentrations [6,8,9]. In turn, this pro-inflammatory status has also been associated with intracellular copper efflux, mainly in the form of ceruloplasmin [28]. Hence, this is finally mirrored in increased blood content of total copper [7,8,9], and especially of cuproproteins [29,30], in agreement with our size-fractionation findings. This hypothesis linking inflammation with alterations in the copper-zinc ratio was corroborated by correlation analysis (Figure 1A,B), as reflected by the positive association found between plasma copper, in both total and HMM fractions, and multiple inflammatory markers (e.g., white blood cell-based inflammatory indices). Correlation analyses also supported close inter-relationships between copper and other markers of oxidative stress and dyslipidemia. As a redox-active metal, elevated copper in circulation may contribute to exacerbate oxidative stress by generating free radical species [28], thereby inducing lipid peroxidation (i.e., TBARS production) and impairing the proper functioning of antioxidant defenses (i.e., reduced catalase activity). In this context, previous studies also suggest that systemic inflammation and oxidative stress serve as pathophysiological mediators in the abnormal lipid profiles associated with copper overload [31]. In contrast, plasma zinc metalloproteins were negatively correlated with mean glucose levels along the OGTT and positively with the WBISI score (Figure 1B), probably as a reflection of the crucial involvement of this trace element in proper insulin production, storage, and action [32].

Nevertheless, it should be noted here that controversial findings have previously been published regarding the link between copper status and metabolic disorders, with some studies reporting lower levels of this metal in subjects with obesity [33] and related comorbidities, such as non-alcoholic fatty liver disease [34,35]. In this respect, is has been described that copper bioavailability is age-dependent [36], so differences in demographic characteristics might account in a large extent for the inconsistencies found in the literature. This further reinforce the need of using well-characterized cohorts and properly addressing inter-individual variability factors.

### 4.2. Circulating Iron Deficiency

Children with obesity had lower ferroprotein levels, and the same trend was observed for the total iron content without reaching statistical significance, as described by other authors [6,7,9]. Interestingly, these alterations were only detected among IR subjects, whereas children with metabolically healthy obesity showed similar iron status than controls. Although there are various hypotheses regarding the origin of iron deficiency in obesity (e.g., inadequate iron intake, raised iron requirements due to higher body mass, decreased muscle myoglobin due to low physical activity), the most likely explanation revolves around the involvement of hepcidin, an important regulator of iron metabolism [37]. Under chronic inflammation, cytokines promote the release of hepcidin from adipose tissue and liver, which inhibits intestinal iron absorption and its efflux from the major iron-transporting tissues. This results in iron sequestration as cytoplasmatic ferritin, thereby reducing circulating concentrations. In agreement with this, we found that plasma iron negatively correlated with several inflammatory markers, but also with hyperglycemia and hyperinsulinemia-related variables (Figure 1A,B). In this respect, it has been reported that hyperinsulinemia provokes the accumulation of iron into pancreatic β cells, which triggers the formation of radical oxygen species, causes mitochondrial dysfunction, and subsequently impairs insulin secretion [38]. In addition to β cells, iron overload in adipocytes also disrupts insulin signaling, leading to decreased insulin sensitivity [39]. This could explain our observation that circulating iron levels were only altered among ObIR+ individuals.

### 4.3. The Role of Selenium and Manganese in Disturbances of the Antioxidant Defense

The decreases observed in the plasma levels of selenium and manganese in the HMM fraction (Table 2), but not in their total contents nor within LMM species, pinpoint the involvement of Se/Mn-dependent proteins in childhood obesity. As previously reported, children with obesity are characterized by lower concentrations of selenium in blood [6,9], manganese in hair [13] and teeth [5], and reduced activity of related antioxidant enzymes, such as glutathione peroxidase [19,40] and manganese superoxide dismutase [41]. These disturbances have been attributed to the characteristic pro-oxidant status underlying obesity, which increases the demand for endogenous antioxidants. In turn, this exacerbated oxidative stress is known to be closely inter-related to other pathogenic hallmarks behind childhood obesity, as evidenced by the negative associations computed between these elements and other variables related to glucose control and inflammation (Figure 1A,B). Regarding carbohydrate metabolism, the proper homeostasis of selenium and manganese has been proved to be essential for mitigating oxidative damage processes that perturb insulin secretion in pancreatic β cells [32]. Moreover, both essential elements have also been found to alleviate inflammatory signaling pathways [40,42]. To conclude, it should be noted that selenium showed a consistent positive correlation with all cholesterol fractions (i.e., TC, LDL-C, HDL-C), plausibly because of the central role that isopentenyl pyrophosphate, an intermediate in the cholesterol biosynthetic pathway, plays in the synthesis of selenoproteins [43].

### 4.4. Chromium, Cobalt, and Molybdenum Regulate Glucose and Lipid Metabolism

A substantial reduction in the contents of metalloproteins containing chromium, cobalt, and molybdenum was observed in the plasma from children with metabolically unhealthy obesity, and to a lesser extent in the ObIR- group, compared to controls (Table 2). Similarly, LMM chromium species showed the same downward trend among children with obesity without reaching statistical significance. This is in accordance with studies reporting lower cobalt blood levels in children with obesity [6,10], whereas the available literature describes negative associations between obesity and circulating chromium [44,45] and molybdenum [46,47] only within adult populations. The main biological functions of chromium, cobalt, and molybdenum revolve around their participation in the regulation of glucose and lipid metabolism. Chromium has a positive effect on blood glucose control by improving insulin signaling through the activation of the tyrosine kinase receptor and by stimulating the translocation of the protein glucose transporter 4 in adipocytes, whereas its supplementation has been demonstrated to prevent atherogenic dyslipidemia through a myriad of mechanisms that remain unclear (e.g., enhanced β-oxidation, cholesterol synthesis inhibition, expression of peroxisome proliferator-activated receptors) [48]. Molybdenum, as a cofactor of several enzymes involved in central metabolic pathways, may also enhance glucose-induced insulin secretion, upregulate insulin signal transduction, inactivate glycogen synthase, and alleviate lipid peroxidation and lipid accumulation [32]. Likewise, cobalt also plays essential roles in glucose and lipid metabolisms by improving tolerance to glucose, regulating glycogen depots via suppressing glucagon signaling, and ameliorating dyslipidemia [49,50]. Altogether, this is in line with the results from our correlation analyses that showed negative associations with markers of hyperglycemia (i.e., mean and AUC values for glucose), hyperinsulinemia (i.e., fasting insulin, HOMA-IR), and dyslipidemia (i.e., CRI-I, LDL-C, TC), and positive correlations with variables related to healthy insulin sensitivity (i.e., WBISI) and lipid profile (i.e., HDL-C) (Figure 1A,B). In particular, it should be noted here that the major active form of chromium in the organism is the low-molecular-weight chromium-binding substance, which explains the strong negative correlation found between LMM-Cr species, AUC_Glc_, CRI-I, and obesity parameters (Figure 1C). Furthermore, because of their protective role against dyslipidemia, the three metals were also negatively correlated with plasma CRP levels, a classical cardiovascular risk factor that may play a direct role in atherogenesis. However, HMM chromium and cobalt species were curiously associated with glycated hemoglobin in a positive fashion, which deserves further investigation.

### 4.5. Accumulation of Metalloproteins in Erythrocytes

Unlike the results reported for plasma, the analysis of erythrocyte samples revealed significant increases in HMM species of different elements, namely iron, copper, selenium, and to a lesser extent zinc and manganese, in the erythroid cytosolic fraction of children with obesity (Table 2). The most plausible explanation for these findings could be attributed to perturbations in metal transport across biological compartments. As described under Section 4.2, inflammation may inhibit cell iron efflux via hepcidin induction, but also promotes its uptake through the expression of different transporters, including transferrin receptors and the divalent metal transporter 1 [37]. Because of their non-specific nature, the upregulation of these metal transporters could be responsible not only for the erythroid sequestration of iron, but also of other metals such as copper, zinc, and manganese. As a reflection of the mediation of inflammation in these multi-elemental alterations, erythrocyte HMM metal species showed consistent positive correlations with multiple inflammatory markers (Figure 2A,B). Correlation analyses also suggested that the accumulation of redox-active metals could be related to oxidative stress mechanisms and impaired glucose metabolism. Surprisingly, however, we found that total and HMM-bound erythrocyte levels of iron, copper, and zinc negatively correlated with the TBARS content, whereas the association was positive with protein carbonyls. In this vein, it is unclear if erythroid HMM metals species directly participate in the induction of oxidative stress, or whether their accumulation within erythrocytes is part of a defensive mechanism aimed to buffer circulating metal levels for minimizing oxidative damage. In addition to the above-mentioned elements, erythrocytes from children with obesity also had elevated concentrations of selenoproteins, probably as a compensatory mechanism to balance the pro-oxidant status provoked by intracellular metal overload. This hypothesis was supported by the positive correlation found between erythroid selenium, in both total and HMM fractions, and catalase activity, a protective enzyme involved in the antioxidant defense (Figure 2A,B).

### 4.6. The Importance of the Labile Metal Pool

Although elements conforming the LMM fraction were not significantly different between the study groups, the results from correlation analyses emphasized the importance that labile metals may have in the pathogenic events underlying childhood obesity. Under normal conditions, circulating metals are predominantly coordinated with proteins, but aberrant failures in their homeostasis may provoke the release of free species. This pathological condition has not been described to date in obesity, but it has been extensively investigated in Alzheimer’s disease, where increased content of labile iron and copper has been associated with profound impairments in the proteins that regulate their trafficking, including decreased ability of ferritin to retain iron, disturbed transferrin-mediated transport, and ceruloplasmin fragmentation [25]. In this respect, we found in the present study that many of the LMM metal species detected in plasma positively correlated with transferrin saturation (Fe, r = 0.53; Zn, r = 0.36; Mn, r = 0.32), which point to transferrin-mediated metal transport as a plausible source of circulating free species in childhood obesity. This labile pool of redox-active metals may in turn have toxic repercussions on health, particularly by inducing oxidative stress (Figure 1C). However, contrary to the results observed for the total and HMM fractions, labile metal species showed a consistent negative association with deregulations in glucose and lipid metabolism, thereby highlighting the utmost importance of the chemical form in which metals are present in their final biological activity.

## 5. Conclusions

The present study represents the first comprehensive investigation of the plasmatic and erythroid multi-elemental biodistribution in childhood obesity. The main strength of our work lies in the use of a well-characterized cohort to assess the role of metal homeostasis in the crosstalk between childhood obesity and IR, as well as to associate circulating metal alterations with other pathogenic hallmarks, such as inflammation, oxidative stress, impaired glucose metabolism, and dyslipidemia. In this respect, we found that metal-related abnormalities were sharpened in subjects presenting IR compared to children with metabolically healthy obesity, thereby emphasizing the importance of addressing within-group differences driven by the presence of concomitant comorbidities. Furthermore, the application of a high-throughput method for size-fractionation of metal species enabled us to obtain a deeper understanding of the mechanisms that may underlie metal disturbances in childhood obesity. In this respect, our results revealed that major perturbances are observed at the metalloproteome level, although labile metals could also play a pivotal role, which deserves further investigation. These discrepancies depending on the fraction under investigation highlight the crucial importance of characterizing metal species instead of only determining total metal contents, and could explain, at least in part, the inconsistencies repeatedly reported in the literature, especially for minor elements. Another strength of this work is the study, for the first time, of the multi-elemental alterations occurring in the erythrocyte, which is of great interest considering the potential of these blood cells as systemic indicators of the metabolic and redox status of the organism. Interestingly, we found that children with obesity tended to accumulate a wide range of metalloproteins in the erythroid cytoplasmatic fraction, which could be indicative of abnormal metal transport across biological compartments. However, some limitations deserve to be mentioned as well, such as the relatively small sample size of the study population and the lack of an independent cohort for validation purposes. Accordingly, future studies in larger populations are needed to further validate our findings and better characterize the involvement metals in obesity. In particular, the stratified characterization of obesity according to the presence or absence of comorbidities seems to be crucial to facilitate the development of more effective diagnostic, preventive, and intervention approaches in the context of precision medicine.

## Figures and Tables

**Table 1:** Demographic, anthropometric, and biochemical characteristics of the study population. Results are expressed as mean ± standard deviation (except for sex, expressed as percentage). NS, non-significant.

	CNT	ObIR−	ObIR+	*p*-Value
Demographics and anthropometry				
*n*	26	15	31	NS
Age (years)	8.6 ± 1.6	9.3 ± 1.6	9.5 ± 1.4	NS
Sex (% male)	61.5	60.0	54.8	NS
Weight (kg)	27.6 ± 5.1 ^a^	57.5 ± 13.4 ^b^	59.7 ± 10.4 ^b^	7.0 × 10^−24^
Body mass index (BMI, kg/m^2^)	16.5 ± 1.5 ^a^	28.7 ± 3.5 ^b^	29.4 ± 4.3 ^b^	2.0 × 10^−24^
Waist circumference (WC, cm)	58.4 ± 5.8 ^a^	94.5 ± 13.2 ^b^	94.1 ± 10.4 ^b^	1.1 × 10^−14^
Carbohydrate metabolism				
Fasting glucose (Glc_0_, mg/dL)	83.9 ± 4.4	84.3 ± 4.3	86.8 ± 8.4	NS
Mean glucose (MeanGlc, mg/dL)	-	104.9 ± 8.1 ^a^	129.1 ± 27.6 ^b^	4.9 × 10^−2^
Area under the curve for glucose (AUC_Glc_, mg·h/dL)	-	182.6 ± 38.5	215.3 ± 61.9	NS
Fasting insulin (Ins_0_, µU/mL)	4.6 ± 2.1 ^a^	12.6 ± 3.4 ^b^	19.9 ± 10.0 ^b^	2.1 × 10^−8^
Mean insulin (MeanIns, µU/mL)	-	53.9 ± 20.4 ^a^	126.7 ± 57.2 ^b^	3.6 × 10^−3^
Area under the curve for insulin (AUC_Ins_, µU·h/mL)	-	125.2 ± 49.7 ^a^	266.0 ± 138.6 ^b^	1.2 × 10^−2^
HOMA-IR	1.0 ± 0.4 ^a^	2.6 ± 0.7 ^ab^	4.4 ± 2.6 ^b^	1.6 × 10^−3^
WBISI	-	0.5 ± 0.2 ^a^	0.3 ± 0.2 ^b^	1.3 × 10^−2^
Glycated hemoglobin (HbA1c, %)	5.2 ± 0.2 ^a^	5.3 ± 0.3 ^b^	5.3 ± 0.3 ^b^	7.5 × 10^−3^
Lipid metabolism				
Total cholesterol (TC, mg/dL)	165.8 ± 27.0	160.0 ± 29.5	160.5 ± 35.4	NS
Low-density lipoprotein cholesterol (LDL-C, mg/dL)	92.1 ± 23.2	96.0 ± 25.6	99.4 ± 32.3	NS
High-density lipoprotein cholesterol (HDL-C, mg/dL)	66.0 ± 13.7 ^a^	47.2 ± 9.4 ^b^	43.8 ± 7.9 ^b^	4.0 × 10^−8^
Castelli risk index-I (CRI-I)	2.6 ± 0.5 ^a^	3.4 ± 0.8 ^b^	3.8 ± 0.9 ^c^	9.4 × 10^−8^
Triglycerides (TG, mg/dL)	48.2 ± 19.6 ^a^	79.6 ± 34.1 ^b^	91.0 ± 46.1 ^b^	4.0 × 10^−6^
Inflammatory markers				
C-reactive protein (CRP, mg/L)	0.6 ± 0.4 ^a^	3.8 ± 0.1 ^b^	5.1 ± 7.0 ^b^	1.3 × 10^−6^
Systemic immune inflammation index (SII)	314.6 ± 182.6 ^a^	453.9 ± 198.8 ^b^	512.5 ± 297.8 ^b^	6.0 × 10^−3^
Systemic inflammation response index (SIRI)	0.6 ± 0.3 ^a^	0.8 ± 0.3 ^ab^	1.1 ± 0.9 ^b^	5.2 × 10^−3^
Aggregate index of systemic inflammation (AISI)	172.9 ± 103.7 ^a^	269.4 ± 124.1 ^b^	357.2 ± 312.9 ^b^	1.5 × 10^−3^
Platelet-to-lymphocyte ratio (PLR)	123.5 ± 52.7	126.2 ± 39.3	125.2 ± 50.3	NS
Neutrophil-to-lymphocyte ratio (NLR)	1.0 ± 0.5 ^a^	1.3 ± 0.5 ^b^	1.6 ± 0.8 ^b^	1.3 × 10^−3^
Oxidative stress markers				
Thiobarbituric acid reactive substances (TBARS, nmol/mg protein)	2.8 ± 0.7 ^a^	3.6 ± 2.1 ^b^	3.6 ± 1.5 ^b^	4.9 × 10^−2^
Protein carbonyls (PC, nmol/mg protein)	0.3 ± 0.3 ^a^	0.6 ± 0.9 ^b^	0.7 ± 0.6 ^b^	3.5 × 10^−2^
Catalase activity, (CAT, µmol H_2_O_2_/ min·mg protein)	4.2 ± 2.6 ^a^	2.5 ± 1.6 ^b^	2.3 ± 0.8 ^b^	6.4 × 10^−3^

Superscript letters within each row indicate significant differences between groups that are marked with different letters, according to the post-hoc Fisher LSD test (*p* < 0.05).

**Table 2:** Concentrations of metal and metalloid elements in the total, high molecular mass (HMM), and low molecular mass (LMM) fractions from plasma and erythrocyte samples. Results are expressed as mean ± standard deviation (µg/L). ND, non-detected; NS, non-significant.

		Plasma				Erythrocytes			
		CNT	ObIR−	ObIR+	*p*-Value	CNT	ObIR−	ObIR+	*p*-Value
Cadmium	Total	0.0024 ± 0.0021	0.0021 ± 0.0015	0.0031 ± 0.0034	NS	1.8 ± 0.8	1.7 ± 0.7	1.8 ± 0.5	NS
	HMM	0.0022 ± 0.0022	0.0024 ± 0.0020	0.0029 ± 0.0033	NS	1.6 ± 0.6	1.7 ± 0.2	1.7 ± 0.3	NS
	LMM	ND	ND	ND	-	ND	ND	ND	-
Chromium	Total	10.8 ± 11.5	9.1 ± 9.6	8.5 ± 7.9	NS	ND	ND	ND	-
	HMM	9.4 ± 9.0 ^a^	7.1 ± 4.0 ^ab^	5.4 ± 3.9 ^b^	0.026	ND	ND	ND	-
	LMM	1.6 ± 1.5	0.8 ± 0.8	1.1 ± 1.5	NS	ND	ND	ND	-
Cobalt	Total	1.5 ± 0.4	1.5 ± 0.3	1.6 ± 0.6	NS	ND	ND	ND	-
	HMM	0.9 ± 0.5 ^a^	0.7 ± 0.2 ^b^	0.7 ± 0.2 ^b^	0.031	ND	ND	ND	-
	LMM	0.3 ± 0.1	0.3 ± 0.1	0.3 ± 0.2	NS	ND	ND	ND	-
Copper	Total	1295.4 ± 173.5 ^a^	1523.9 ± 252.3 ^b^	1416.2 ± 221.5 ^b^	0.049	557.2 ± 91.8	583.7 ± 97.9	594.1 ± 102.5	NS
	HMM	1155.2 ± 136.5 ^a^	1349.9 ± 178.7 ^b^	1266.4 ± 179.3 ^b^	0.013	588.5 ± 112.5 ^a^	667.8 ± 53.0 ^b^	703.4 ± 49.9 ^b^	0.031
	LMM	25.5 ± 28.1	24.0 ± 19.7	27.4 ± 39.5	NS	1.6 ± 0.4	1.5 ± 0.3	1.7 ± 0.7	NS
Iron	Total	830.3 ± 284.1	835.4 ± 196.9	769.5 ± 252.2	NS	744,477.1 ± 658,92.9	778,357.1 ± 98,434.9	765,489.9 ± 79,762.8	NS
	HMM	720.6 ± 175.1 ^a^	708.3 ± 137.5 ^a^	670.5 ± 196.9 ^b^	0.013	655,373.1 ± 146,698.9 ^a^	715,995.0 ± 13,934.0 ^ab^	813,085.7 ± 67,382.5 ^b^	0.042
	LMM	36.6 ± 19.2	31.0 ± 16.4	27.3 ± 13.2	NS	24.5 ± 12.0	20.1 ± 7.9	20.1 ± 8.9	NS
Lead	Total	0.025 ± 0.0054	0.025 ± 0.0038	0.024 ± 0.0039	NS	61.5 ± 5.4	63.9 ± 5.4	62.4 ± 8.3	NS
	HMM	0.025 ± 0.0065	0.022 ± 0.0031	0.023 ± 0.0048	NS	57.6 ± 15.1	58.8 ± 6.0	60.4 ± 13.2	NS
	LMM	ND	ND	ND	-	1.3 ± 0.3	1.2 ± 0.2	1.3 ± 0.2	NS
Manganese	Total	4.7 ± 5.6	4.6 ± 7.0	4.8 ± 4.6	NS	18.0 ± 14.9	18.7 ± 11.7	17.4 ± 15.0	NS
	HMM	4.4 ± 1.3 ^a^	4.1 ± 0.9 ^a^	3.6 ± 1.0 ^b^	0.031	17.5 ± 15.8	18.0 ± 16.4	18.2 ± 12.6	NS
	LMM	0.3 ± 0.5	0.6 ± 0.6	0.5 ± 0.6	NS	0.7 ± 0.2	0.6 ± 0.2	0.7 ± 0.1	NS
Molybdenum	Total	2.7 ± 0.7	2.9 ± 0.7	2.8 ± 1.0	NS	23.4 ± 5.1	25.9 ± 5.1	20.9 ± 4.5	NS
	HMM	2.9 ± 2.1 ^a^	2.2 ± 0.6 ^ab^	1.9 ± 0.6 ^b^	0.013	18.5 ± 4.9	17.5 ± 6.9	16.2 ± 3.7	NS
	LMM	ND	ND	ND	-	0.2 ± 0.1	0.2 ± 0.1	0.2 ± 0.1	ND
Selenium	Total	133.8 ± 15.9	123.0 ± 15.6	126.1 ± 20.3	NS	153.6 ± 27.8	145.9 ± 29.9	160.3 ± 50.3	NS
	HMM	143.2 ± 23.4 ^a^	130.4 ± 17.1 ^ab^	125.1 ± 19.6 ^b^	0.013	122.0 ± 92.8 ^a^	131.3 ± 98.1 ^a^	167.2 ± 86.3 ^b^	0.047
	LMM	2.4 ± 2.0	2.1 ± 1.1	2.1 ± 1.7	NS	0.2 ± 0.1	0.2 ± 0.1	0.2 ± 0.1	NS
Zinc	Total	830.1 ± 174.8	744.2 ± 102.5	733.4 ± 182.4	NS	9300.1 ± 1905.7	9401.6 ± 1742.4	9423.1 ± 1738.8	NS
	HMM	942.6 ± 489.4 ^a^	711.1 ± 213.9 ^ab^	641.9 ± 231.3 ^b^	0.013	9114.1 ± 1692.1	8950.9 ± 2024.0	10176.9 ± 1418.2	NS
	LMM	32.2 ± 15.3	30.8 ± 13.4	29.5 ± 13.3	NS	63.6 ± 9.2	58.8 ± 5.1	62.4 ± 7.4	NS

Superscript letters within each row indicate significant differences between groups that are marked with different letters, according to the post-hoc Fisher LSD test (*p* < 0.05).

## Data Availability

The datasets used and/or analyzed during the current study are available from the corresponding author on reasonable request.
